# Exploring the relationship between osteoporosis and polycystic ovary syndrome based on bioinformatics

**DOI:** 10.1097/MD.0000000000029434

**Published:** 2022-06-24

**Authors:** Chun-xiao Dang, Ding Wang, Xiao Yu, Peng-fei Liu, Jin-xing Liu

**Affiliations:** a Shandong University of Traditional Chinese Medicine, Jinan, China; b Affiliated Hospital of Shandong University of Traditional Chinese Medicine, Jinan, China.

**Keywords:** bioinformatics, disease interrelationship, osteoporosis, polycystic ovary syndrome

## Abstract

**Background:**

In recent years, clinical studies have found that there is a close relationship between osteoporosis and polycystic ovary syndrome. However, there are few literature on the pathogenesis of osteoporosis and polycystic ovary syndrome. In order to clarify their common pathogenic mechanism and provide potential targets for drugs to regulate them at the same time, bioinformatics methods are used to explore, so as to provide a new direction for the study of the relationship between diseases in the future.

**Methods:**

To screen the targets of osteoporosis and polycystic ovary syndrome by Genecards, Online Mendelian Inheritance in Man databases and Therapeutic Target Database to take the intersection of the two mappings and upload the intersection targets to the STRING database to construct protein-protein interaction network; to screen the core targets by degree value and import them to Metascape database for Gene Ontology and Kyoto Encyclopedia of Genes and Genomes pathway analysis; and finally, to construct the visualization network of core targets and pathways by Cytoscape software. Ethical approval and informed consent of patients are not required because the data used in this study is publicly available and does not involve individual patient data or privacy.

**Results:**

The core targets of polycystic ovary syndrome and osteoporosis were insulin gene, insulin-like growth factor 1, CTNNB1, serine/threonine kinase 1, signal transducer and activator of transcription 3, LEP, etc. The biological processes involved include the regulation of protein phosphorylation, cell proliferation and differentiation, hormone endocrine, reproductive system and skeletal system. The related pathways were concentrated in Foxo signaling pathway, HTLV-I infection, PI3K-AKT signaling pathway, MAPK signaling pathway and AGE-RAGE signaling pathway in diabetic complications.

**Conclusions:**

There is a close relationship between osteoporosis and polycystic ovary syndrome in terms of target and molecular mechanism. This study used bioinformatics to clarify their targets and mechanisms, providing potential targets for drugs to regulate both diseases simultaneously and providing new directions to explore the relationship between the diseases.

## 1 Introduction

Osteoporosis (OP) is a systemic skeletal disease characterized by a decrease in bone density and bone quality, and the associated factors are complex and diverse.^[1]^ Polycystic ovary syndrome (PCOS) is a disease caused by endocrine abnormalities. The National Institutes of Health reported that the incidence of PCOS in women of reproductive age is about 4% to 10%.^[2]^ Osteoporosis and polycystic ovary syndrome are two types of chronic diseases that are closely related, and a study of 11,106 women in Taiwan^[3]^ found an increased incidence of fractures in patients with PCOS compared to healthy women; Piovezan et al^[4]^ in a systematic evaluation of 31,383 women found that PCOS patients tended to have lower bone mineral density and osteocalcin; there is also literature^[5,6]^ suggests an association between metabolic dysfunction and low vitamin D levels in patients with PCOS.

Therefore, it is urgent to clarify the relationship between osteoporosis and polycystic ovary syndrome and to reduce the risk of osteoporosis in patients with PCOS. A bioinformatics approach was applied to integrate the genetic data of osteoporosis and polycystic ovary syndrome to explore the association and provide a theoretical basis for drug intervention in both diseases simultaneously.

## 2 Materials and methods

### 2.1 Osteoporosis – polycystic ovary syndrome related target collection

The “osteoporosis” “ polycystic ovary syndrome” as keywords retrieval Genecards (https://www.genecards.org/),^[7]^ Online Mendelian Inheritance in Man (http://www.omim.org/),^[8]^ Therapeutic Target Database (http://db.idrblab.net/ttd/)^[9]^ database screening of disease related targets. To improve the accuracy, the top 300 target genes with high Relevance Score were selected from the Genecards database, and the results were combined and de-weighted, and then the Uniprot database was used to screen the clearly identified target genes as the final targets for polycystic ovary syndrome and osteoporosis.

### 2.2 Target mapping and protein-protein interaction (PPI) analysis of osteoporosis – polycystic ovary syndrome

The target genes of osteoporosis and polycystic ovary syndrome were imported into the BMK Cloud (http://www.biocloud.net/) platform to obtain the intersecting genes, and the intersecting genes were uploaded to the STRING database (https://string-db.org/cgi/input.pl) to construct a PPI network.^[10]^ Download the PPI network in tsv format and import it into Cytoscape 3.8.0 software to better visualize the protein-protein interaction relationships. Using Network analyzer plug-in, adjust the size and color of the targets according to the node connectivity (degree), and adjust the thickness and color of the connecting lines according to the combined score, and finally select the targets with the degree value ≥18 to make the core target map of osteoporosis – polycystic ovary syndrome.

### 2.3 Gene Ontology (GO), Kyoto Encyclopedia of Genes and Genomes (KEGG) analysis and visualization network of osteoporosis – polycystic ovary syndrome

The target genes of osteoporosis and polycystic ovary syndrome were imported into Metascape database (http://metascape.org/),^[11]^ and set *P* < .01 and enrichment factor > 1.5 for GO and KEGG analysis. The first 40 GO, KEGG pathways of the two diseases were selected and intersected by logP ascending order, and imported into Cytoscape 3.8.0 software to construct the interaction pathway map of osteoporosis – polycystic ovary syndrome.

## 3 Results

### 3.1 Results of target collection for osteoporosis – polycystic ovary syndrome

We obtained 30,020,315 targets related to polycystic ovary syndrome and 3,002,942 targets related to osteoporosis from Genecards, Online Mendelian Inheritance in Man and Therapeutic Target Database, respectively; 2,921,765 effective targets for polycystic ovary syndrome and 281,138 effective targets for osteoporosis were obtained after screening by Uniprot database. A total of 453 effective target genes for polycystic ovary syndrome and 281 effective target genes for osteoporosis were obtained after combined de-duplication.

### 3.2 Target mapping of osteoporosis – polycystic ovary syndrome and results of PPI analysis

**Figure 1:**
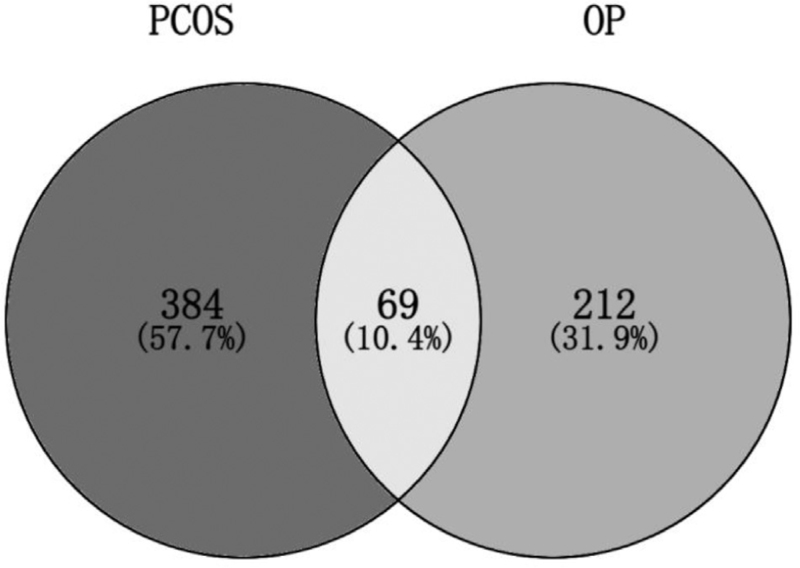
Intersection target of OP and PCOS.

**Figure 2:**
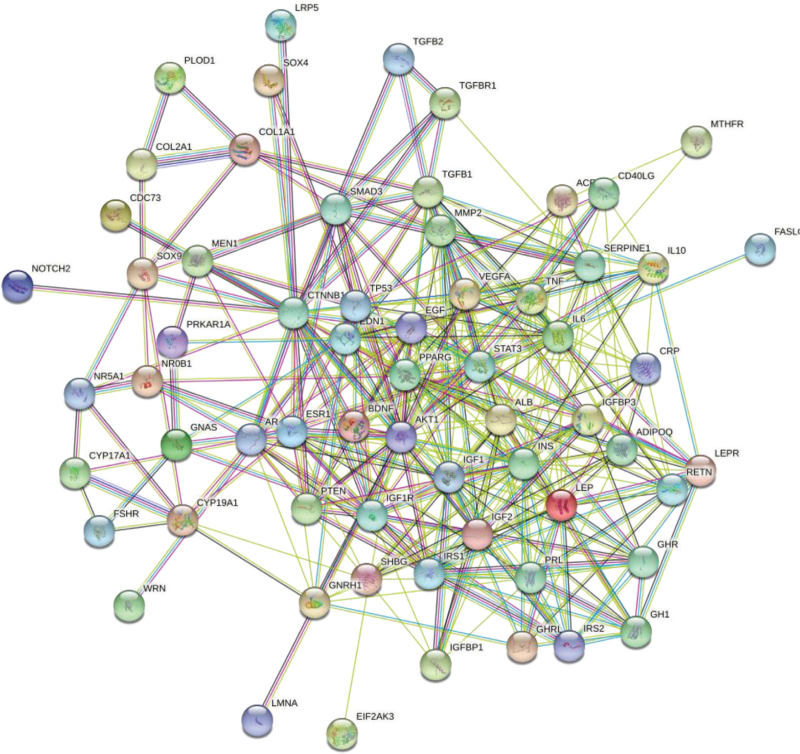
PPI network diagram of intersection targets.

**Figure 3:**
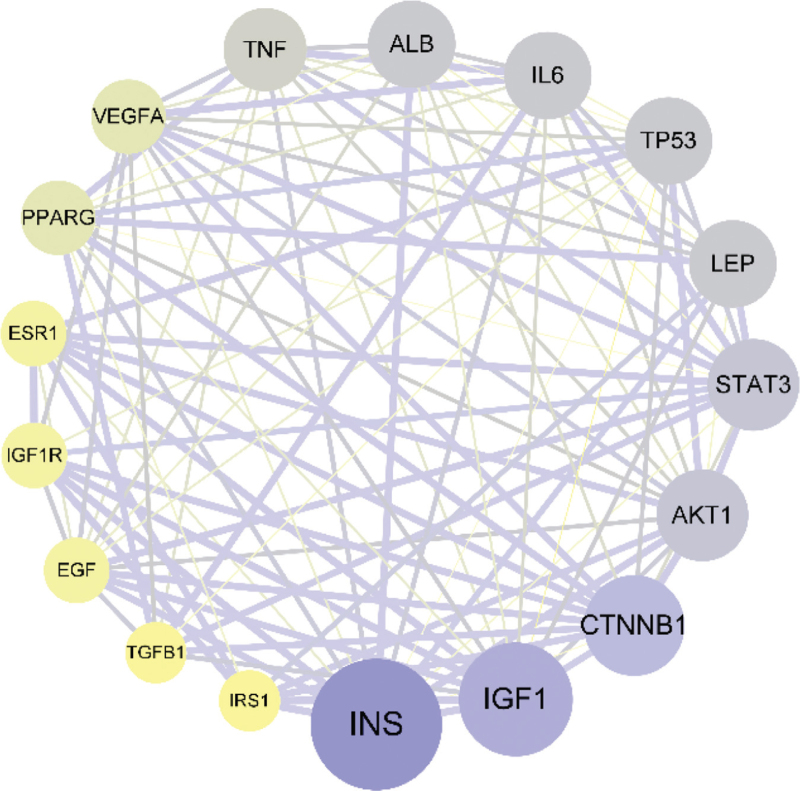
Core target diagram.

Target genes mapping osteoporosis and polycystic ovary syndrome, a total of 70 intersecting genes were obtained (Fig. 1). The intersection genes were uploaded to the STRING database, and the minimum interaction score was set to 0.7 and the free nodes were hidden to generate the PPI interaction network (Fig. 2). The results showed that a total of 69 targets were interlinked, and the network had 363 edges with an average node degree of 10.5 and an average clustering coefficient of 0.552. We downloaded its TSV file and imported into Cytoscape 3.8.0 software, 17 core targets with degree value ≥ 18 were screened (Fig. 3). The obtained core targets directly or indirectly affect osteoporosis and polycystic ovary syndrome.

### 3.3 Results of GO, KEGG analysis in osteoporosis – polycystic ovary syndrome

**Figure 4:**
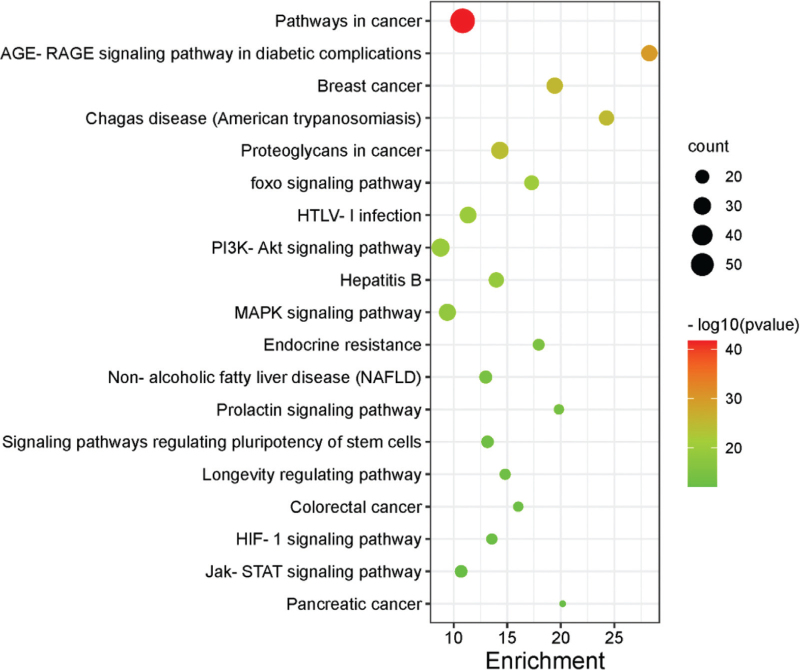
Barplot histogram of signal pathway of OP and PCOS.

**Figure 5:**
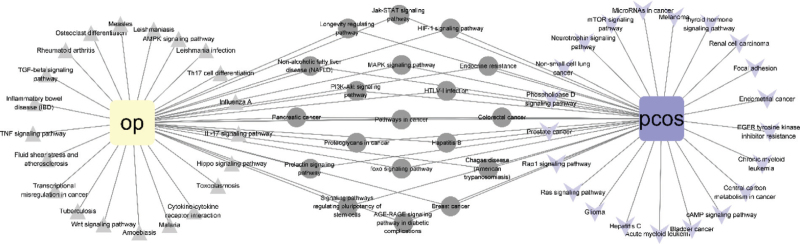
The interaction path between OP and PCOS.

The GO, KEGG signaling pathways of osteoporosis and polycystic ovary syndrome were obtained from the Metascape database, respectively. And the first 40 GO, KEGG pathways of the two diseases were selected in ascending logP order, and finally 17 (GO), 19 (KEGG) interaction pathways were screened (Fig. 4, Table 1, Table 2), and imported into Cytoscape 3.8.0 software to construct the interaction pathway map of osteoporosis – polycystic ovary syndrome (Fig. 5).

**Table 1:** 19 KEGG interaction paths between OP and PCOS.

Gene Ontology	Path description	Log *P*
hsa05200	Pathways in cancer	−41.7856
ko04933	AGE-RAGE signaling pathway in diabetic complications	−29.8879
ko05224	Breast cancer	−25.2937
ko05142	Chagas disease (American trypanosomiasis)	−24.7953
hsa05205	Proteoglycans in cancer	−24.213
hsa04068	Foxo signaling pathway	−20.2934
hsa05166	HTLV-I infection	−19.8598
hsa04151	PI3K-Akt signaling pathway	−19.4415
hsa05161	Hepatitis B	−19.0402
hsa04010	MAPK signaling pathway	−18.3932
hsa01522	Endocrine resistance	−15.1646
ko04932	Non-alcoholic fatty liver disease (NAFLD)	−14.4383
hsa04917	Prolactin signaling pathway	−13.974
hsa04550	Signaling pathways regulating pluripotency of stem cells	−13.0563
hsa04211	Longevity regulating pathway	−12.9737
hsa05210	Colorectal cancer	−12.6346
hsa04066	HIF-1 signaling pathway	−12.3994
hsa04630	Jak-STAT signaling pathway	−12.2563
ko05212	Pancreatic cancer	−12.1551

KEGG = Kyoto Encyclopedia of Genes and Genomes, OP = osteoporosis, PCOS = polycystic ovary syndrome.

**Table 2:** 17 GO interaction paths between OP and PCOS.

Gene Ontology	Path description	Log *P*
GO:0002009	Morphogenesis of an epithelium	−59.0308
GO:0048732	Gland development	−53.7721
GO:0035239	Tube morphogenesis	−52.9034
GO:0048608	Reproductive structure Development	−45.3505
GO:0061458	Reproductive system development	−45.1488
GO:0009725	Response to hormone	−41.8798
GO:0001934	Positive regulation of protein Phosphorylation	−37.9615
GO:0050678	Regulation of epithelial cell proliferation	−37.8068
GO:0001501	Skeletal system development	−37.5617
GO:0043549	Regulation of kinase activity	−37.2621
GO:1901699	Cellular response to nitrogen compound	−37.0043
GO:0071417	Cellular response to organonitrogen compound	−35.7585
GO:1901652	Response to peptide	−33.5401
GO:0008285	Negative regulation of cell population proliferation	−32.9009
GO:0007167	Enzyme linked receptor protein signaling pathway	−31.6865
GO:0045596	Negative regulation of cell differentiation	−30.2734

GO = Gene Ontology, OP = osteoporosis, PCOS = polycystic ovary syndrome.

## 4 Discussion

### 4.1 Results and analysis

Recent studies have found that the two types of diseases, polycystic ovary syndrome and osteoporosis, are interrelated and affect each other.^[12]^ Clinical studies^[13,14]^ PCOS patients were found to have different degrees of bone mineral density loss, which is closely related to leptin resistance and hyperinsulinemia. Therefore, in this study, bioinformatics was used to find the intersection and common pathways in the vast gene network of the two diseases to provide potential targets for drug therapy. A total of 70 intersecting targets were found between polycystic ovary syndrome and osteoporosis, accounting for about 10.38%, with core targets such as insulin gene (INS), insulin-like growth factor 1 (IGF1), CTNNB1, Serine/threonine kinase 1 (AKT1), signal transducer and activator of transcription 3 (STAT3), LEP, etc.; The biological processes involved include the regulation of protein phosphorylation, cell proliferation and differentiation, hormone endocrine, reproductive system and skeletal system; KEGG pathway analysis showed that the Foxo signaling pathway, HTLV-I infection, PI3K-AKT signaling pathway, MAPK signaling pathway and AGE-RAGE signaling pathway in diabetic complications and many other signaling pathways.

As an endocrine disease with complex etiology, polycystic ovary syndrome is closely related to obesity and insulin resistance, and the INS is also considered to be one of the candidate genes for the development of PCOS.^[15]^ The INS is also considered as one of the candidate genes for PCOS. Studies^[16,17]^ have confirmed that decreased insulin receptor activity and PI-3K activity lead to the decrease of glucose uptake rate. Therefore, PCOS patients are prone to metabolic abnormalities such as obesity and decreased glucose tolerance. Meanwhile, the high glucose environment alters osteoblast activity and induces osteoblast apoptosis, leading to reduced bone mineral density and increased fracture risk.^[18,19]^ IGF1, an osteogenic differentiation factor, upregulates the expression of protein kinase and alkaline phosphatase mesenchymal stem cells and participates in cartilage anabolism and repair.^[20]^ Animal experiments^[21,22]^ found that different concentrations of IGF1 play a positive role in regulating the expression of bone genes in cartilage layer and subchondral bone, and the bone thickness of IGF1 knockout mice is significantly lower than that of normal mice, which confirmed the importance of IGF1 in bone growth; meanwhile, the occurrence of hyperandrogenism in PCOS is closely related to hyperinsulinemia, 44–77% of PCOS patients can be combined with hyperinsulinemia, and the higher insulin stimulates IGF1, promotes the release of LH, and enhances the activity of 17α-hydroxylase in follicular membrane cells, which increases the synthesis and secretion of androgens, thus causing the development of hyperandrogenemia^[23]^; AKT1 is an isomer of Akt, mediates many downstream pathways regulated by PI3K,^[24]^ regulates the growth of follicles, the proliferation of granulosa luteal cells and the dynamic balance between bone formation and bone resorption. Some studies^[25]^ speculate that the high expression of AKT1 is one of the reasons affecting the quality of follicles and the function of granulosa luteal cells in patients with PCOS. There are also literature^[26,27]^ reported that activated AKT1 can control the proliferation and differentiation of osteoblasts and osteoclasts through the RANK/RANKL signaling pathway and PI3K/AKT signaling pathway to affect bone metabolism, and Mukherjee et al^[28]^ also found that osteoblast formation was significantly inhibited by culturing AKT1 knockout bone marrow stromal cells or mesenchymal stem cells. The STAT3 dimer formed by the dimerization of STAT3 can activate STAT,^[29]^ promote the transcription of STAT3 target genes, form JAK2/STAT3 signal pathway mediated by IL-6,^[30]^ inhibit the expression of TNF, VEGF and other inflammatory factors, and improve leptin resistance in PCOS patients.^[31]^ Meanwhile, STAT3 dimer can also promote the proliferation and differentiation of Th17 cells, promote the secretion of IL-17 and TNF-α.^[32]^ It affects the formation of osteoclasts and bone resorption process, and inhibits the apoptosis of osteoblasts by promoting the expression of apoptosis inhibitory protein Bcl-2.^[33]^

Foxo signaling pathway, as one of the pathways mediating the inflammatory response, can improve insulin resistance, regulate glucose metabolism in PCOS patients.^[34]^ It can prevent the effect of high glucose environment on osteoblast apoptosis, and regulate the free oxygen concentration in osteoblasts to promote osteoblast differentiation.^[35]^ It can also reduce the damage to cartilage caused by inflammation. He Tingting et al^[36]^ found that the level of anti-apoptotic gene Foxo1 in PCOS patients was lower than that in normal women, suggesting that the low expression of Foxo may be one of the reasons for ovarian granulosa cell autophagy and follicular development disorder in PCOS patients. As an important pathway to regulate cell proliferation, differentiation, metabolism and other basic functions, PI3K/AKT pathway can regulate the function of ovarian granulosa cell, affect hormone levels in vivo, and also affect bone metabolism by controlling the proliferation and differentiation of osteoblasts and osteoclasts, and is a key pathway to affect the functional coordination between osteoblasts and osteoclasts.^[27]^ Mei Shao et al^[37]^ found that PI3K/AKT signaling pathway could inhibit apoptosis and autophagy of ovarian granulosa cells in PCOS patients, promote cell differentiation and proliferation, and improve polycystic ovary syndrome. Animal experiments^[38]^ demonstrated that activation of PI3K/AKT signaling pathway can bidirectionally regulate osteoblast differentiation and osteoclast apoptosis. Dina et al^[39]^ and Tao Ling et al^[40]^ found that the activation of PTHrP gene in HTLV-I infected patients can affect bone mineral deposition, promote osteolysis and cause osteoporosis. MAPK signaling pathway is an important pathway that regulates the proliferation and differentiation of osteoclasts and osteoblasts, and can also promote the osteogenic differentiation of bone marrow mesenchymal stem cells (BMSCs), accelerate calcium deposition, and prevent the occurrence of osteoporosis.^[41]^ The high insulin status of PCOS patients leads to abnormal activation of MAPK signaling pathway, which affects the growth and differentiation of ovarian granulosa cells. Xu Jinbang et al^[42]^ found that the artificial cycle of acupuncture and drugs can regulate the mitotic activity of ovarian granulosa cells through MAPK signaling pathway, and improve the reproductive endocrine disorders of PCOS patients.

## 5 Conclusions

In this study, we searched for common targets and pathways of action between polycystic ovary syndrome and osteoporosis through bioinformatics, and expressed the interrelationship between them more clearly through visualization network, which also provides potential targets for drugs to regulate both diseases simultaneously. It is found that insulin resistance, aggravation of glucose metabolism disorder and imbalance of hormone level in polycystic ovary syndrome will affect the proliferation and differentiation of osteoblasts and osteoclasts. Foxo signal pathway, PI3K/AKT signal pathway and JAK2/STAT3 signal pathway all play a direct or indirect role. Of course, with the continuous development of bioinformatics and disease databases, the reliability and accuracy of data will continue to improve, and further studies are needed to make the conclusions more reliable.

## Author contributions

**Conceptualization:** Chun-xiao Dang, Jin-xing Liu.

**Data curation:** Chun-xiao Dang, Ding Wang, Xiao Yu.

**Formal analysis:** Chun-xiao Dang, Ding Wang.

**Funding acquisition:** Peng-fei Liu, Jin-xing Liu, Xiao Yu.

**Investigation:** Chun-xiao Dang, Peng-fei Liu.

**Methodology:** Chun-xiao Dang, Yu Xiao.

**Writing – original draft:** Chun-xiao Dang, Ding Wang.

**Writing – review & editing:** Peng-fei Liu, Jin-xing Liu.

## References

[1] Alejandro P Constantinescu F. A review of osteoporosis in the older adult: an update. Rheum Dis Clin North Am 2018;44:437–51. 30001785 10.1016/j.rdc.2018.03.004

[2] Azziz R Woods KS Reyna R. The prevalence and features of the polycystic ovary syndrome in an unselected population. J Clin Endocrinol Metabol 2004;89:2745–9. 10.1210/jc.2003-032046 15181052

[3] Yang HY Lee HS Huang WT. Increased risk of fractures in patients with polycystic ovary syndrome: a nationwide population-based retrospective cohort study. Bone Miner Metab 2018;36:741–8. 10.1007/s00774-017-0894-8 29280078

[4] Piovezan JM Premaor MO Comim FV. Negative impact of polycystic ovary syndrome on bone health: a systematic review and meta-analysis. Hum Reprod Update 2019;25:633–45. 31374576 10.1093/humupd/dmz020

[5] Di BF Catalano A Bellone F. Vitamin D, bone metabolism, and fracture risk in polycystic ovary syndrome. Metabolites 2021;11:116. 33670644 10.3390/metabo11020116 PMC7922814

[6] Muscogiuri G Altieri B Annweiler C. Vitamin D and chronic diseases: the current state of the art. Arch Toxicol 2017;91:97–107. 27425218 10.1007/s00204-016-1804-x

[7] Stelzer G Rosen R Plaschkes I. The genecards suite: from gene data mining to disease genome sequence analysis. Curr Protoc Bioinform 2016;54:01–33. 10.1002/cpbi.5 27322403

[8] Joanna JS Hamosh A. Searching online Mendelian inheritance in man (OMIM): a knowledgebase of human genes and genetic phenotypes. Curr Protoc Bioinform 2017;58:01–12. 10.1002/cpbi.27 PMC5662200 28654725

[9] Wang YX Zhang S Li FC. Therapeutic target database 2020: enriched resource for facilitating research and early development of targeted therapeutics. Nucl Acids Res 2020;48:D1031–41. 31691823 10.1093/nar/gkz981 PMC7145558

[10] Szklarczyk D Gable AL Lyon D. STRING v11: protein protein association networks with increased coverage, supporting functional discovery in genome-wide experimental datasets. Nucl Acids Res 2019;47:D607–13. 30476243 10.1093/nar/gky1131 PMC6323986

[11] Zhou Y Zhou B Pache L. Metascape provides a biologist oriented resource for the analysis of systems-level datasets. Nat Commun 2019;10:1523. 30944313 10.1038/s41467-019-09234-6 PMC6447622

[12] Krishnan A Muthusami S. Hormonal alterations in PCOS and its influence on bone metabolism. J Endocrinol 2017;232:R99–113. 27895088 10.1530/JOE-16-0405

[13] Abdulameer SA Syed Sulaiman SA Hassali MA. Osteoporosis and type 2 diabetes mellitus: what do we know, and what we can do. Patient Prefer Adherence 2012;435–48. 22791981 10.2147/PPA.S32745 PMC3393120

[14] Gao MY Huang HJ. Analysis of factors affecting bone density changes in polycystic ovary syndrome. Southeast Def Med 2016;18:405–7.

[15] Liu X Yu CJ Liu XM. Etiology and diagnostic criteria of polycystic ovary syndrome. J Pract Obstetr Gynecol 2018;34:561–4.

[16] Romina F Paulina O Carlos R. Changes in the expression of insulin signaling pathway molecules in endometria from polycystic ovary syndrome women with or without hyperinsulinemia. Mol Med 2010;16:129–36. 20011249 10.2119/molmed.2009.00118 PMC2792869

[17] Anhui Medical University, Wei ZL. Effects of Metformin on Insulin Receptor Substrate-1 and P450 Aromatase in Ovarian Granulosa Cells of PCOS Patients. 2011.

[18] Ruppert K Cauley J Lian Y. The effect of insulin on bone mineral density among women with type 2 diabetes: a SWAN pharmacoepidemiology study. Osteoporos Int 2018;29:347–54. 29075805 10.1007/s00198-017-4276-9 PMC5818624

[19] Wang QQ Zhang BB Xu YL. The relationship between serum osteocalcin concentration and glucose metabolism in patients with type 2 diabetes mellitus. Int J Endocrinol 2013;2013:842598. 23533407 10.1155/2013/842598 PMC3603198

[20] Yang ZQ Zhang HL Duan CC. IGF1 regulates RUNX1 expression via IRS1/2: Implications for antler chondrocyte differentiation. Cell Cycle 2017;16:522–32. 28055425 10.1080/15384101.2016.1274471 PMC5384582

[21] Zhang Z Li L Yang W. The effects of different doses of IGF-1 on cartilage and subchondral bone during the repair of full-thickness articular cartilage defects in rabbits. Osteoarthr Cartil 2017;25:309–20. 10.1016/j.joca.2016.09.010 27662821

[22] Matilda H-C Sheng Zhou XD. Disruption of the insulin-like growth factor-1 gene in osteocytes impairs developmental bone growth in mice. Bone 2013;52:133–44. 23032105 10.1016/j.bone.2012.09.027

[23] Rosenfield RL Ehrmann DA. The pathogenesis of Polycystic Ovary Syndrome (PCOS): The hypothesis of PCOS as functional ovarian hyperandrogenism revisited. Endocr Rev 2016;37:467–520. 27459230 10.1210/er.2015-1104 PMC5045492

[24] Gan CY Zheng ZW Liang BJ. Exploring the mechanism of action of Garcinia cambogia total flavonoids against hepatocellular carcinoma from PI3K/AKT/p53 pathway. Chin J Exp Formul 2019;25:90–6.

[25] Nekoonam S Naji M Nashtaei MS. Expression of AKT1 along with AKT2 in granulosa-lutein cells of hyperandrogenic PCOS patients. Arch Gynecol Obstet 2017;295:1041–50. 28271235 10.1007/s00404-017-4317-9

[26] Adapala NS Barbe MF Tsygankov AY. Loss of Cbl-PI3K interaction enhances osteoclast survival due to p21-Ras mediated PI3K activation independent of Cbl-b. J Cell Biochem 2014;115:1277–89. 24470255 10.1002/jcb.24779 PMC4634568

[27] Chen YH Gong ZQ Cui L. The role of PI3K/AKT signaling pathway in the pathological process of osteoporosis. Chin J Osteoporos 2015;21:356–60.

[28] Mukherjee A Rotwein P. Selective signaling by Akt1 controls osteoblast differentiation and osteoblast-mediated osteoclast development. Mol Cell Biol 2012;32:490–500. 22064480 10.1128/MCB.06361-11 PMC3255772

[29] May P Schniertshauer U Gerhartz C. Signal transducer and activator of transcription STAT3 plays a major role in gp130-mediated acute phase protein gene activation. Acta Biochim Pol 2003;50:595–601. 14515142

[30] Atreyee B Yogamaya DP Abilash VG. Role of IL-6 signalling in polycystic ovarian syndrome associated inflammation. J Reprod Immunol 2020;141:103–55. 10.1016/j.jri.2020.103155 32526588

[31] Lv LQ Li W. Expression and phosphorylation of leptin signaling molecules-JAK2 and STAT3 proteins in adipose tissue of patients with polycystic ovary syndrome combined with insulin resistance. China Mater Child Health 2016;31:3922–5.

[32] Li Y Lu L Xie Y. Interleukin-6 knockout inhibits senescence of bone mesenchymal stem cells in high-fat diet-induced bone loss. Front Endocrinol (Lausanne) 2021;11:622–950. 10.3389/fendo.2020.622950 PMC7933660 33679606

[33] Jiao J Wang Y Sun X. Midazolam induces A549 cell apoptosis in vitro via the miR-520d-5p/STAT3 pathway. Int J Clin Exp Pathol 2018;11:1365–73. 31938232 PMC6958164

[34] Bao QQ Li MR Huang R. Isorhamnetin regulates AKT-FOXO1 pathway to improve the mechanism of glucose metabolism in insulin-resistant HepG2 cells. Food Ind Sci Technol 2020;41:320–4.

[35] Second Military Medical University, He YJ. Mechanism of action and in vivo metabolic study on the protection of oxidatively damaged osteoblasts by Cynaroside through FoxO1. 2015.

[36] He TT Liu X Liu HB. Expression and significance of granulosa cell forkhead box protein O1 mRNA in patients with polycystic ovary syndrome. J Shandong Univ (Med Ed) 2019;57:75–9.

[37] Shao M Wang JC. Effects of Epimedium on apoptosis and autophagy of granulosa cells in polycystic ovary syndrome. Chin J Clin Pharmacol 2021;37:2830–3.

[38] Xi JC Zhang HY Guo LX. The PI3K/AKT cell signaling pathway is involved in regulation of osteoporosis. J Recep Sig Trans Res 2015;35:640–5. 10.3109/10799893.2015.1041647 26390889

[39] Dina S Luis C Arturo B. Osteoporosis in HTLV-I-associated myelopathy/tropical spastic paraparesis (HAM/TSP). Bone 2003;33:192–6.14499352

[40] Yang TAOL Wang LH. Research progress of human T-cell leukemia virus type 1/human T-cell-loving virus type 1 (HTLV-1) and its relationship with related diseases. J Cell Mol Immunol 2019;35:89–94.

[41] Wang JB Chen YP Zhang XY. Progress of research on Epimedium-mediated MAPK signaling pathway for the prevention and treatment of osteoporosis. Chin J Osteoporos 2021;27:1651–5.

[42] Xu JB Yang JJ You XM. Effect of needle-medicine manual cycle therapy on MAPK/ERK pathway in patients with phlegm-damp polycystic ovary syndrome. Chin J Integr Med 2018;38:415–20.

